# Metabolomics analysis reveals diverse nematicidal metabolites from spore-forming bacteria

**DOI:** 10.3934/microbiol.2026009

**Published:** 2026-04-23

**Authors:** Ziqiang Zheng, Yonghong Zhou, Pengxi Cao, Lingling Gong, Chaofan Zheng, Yujiao Huang, Yu Li, Wenjing Le, Jinliang Zhang, Xianwei Zhou, Jing Tan, Chenfei Guo, Yuanxiang Xu, Chang Chen, Ningchang Yang, Luqing Li, Yuhan Sun, Shen-Cai Hu, Fang Peng, Luhong Sun, Shuang Yu, Hongxun Wang

**Affiliations:** 1 School of Life Science and Technology, Wuhan Polytechnic University, Wuhan 430023, China; 2 Wuhan Qianmo Bio-agriculture Technology Co., Ltd., Medicine Garden, Miaoshan Development Zone, Jiangxia District, Wuhan 430299, China; 3 Hubei Enshi Selenium-rich Resource Observation and Research Station, Enshi, 445023, China; 4 Key Laboratory of Biodiversity and Environment on the Qinghai-Tibet Plateau, Ministry of Education, School of Ecology and Environment, Tibet University, Lhasa, 850000, Tibet, People's Republic of China

**Keywords:** Plant-parasitic nematodes, spore-forming bacteria, metabolites, peptides

## Abstract

Plant-parasitic nematodes are very important plant pathogens that can damage almost all crops worldwide. There has been a progressive decline in the use of chemical compounds because of their high toxicity to humans, livestock, and the environment. Spore-forming Bacilli as excellent biological agents are an effective and eco-friendly solution to control nematodes. However, our previous study showed that genomes of some highly nematicidal strains contain less virulent factor genes. Metabolomics studies based on mass spectrometry (MS) have shown that spore-forming strains can produce five classes of nematicidal secondary metabolites, including macrolide compounds (MCs; selamectin), triazines (Ts; prometon), piprazine derivatives (PDs; diethylcarbamazine), benzene and six-membered heterocyclic compounds (BSMHCs; crotamiton, amodiaquine and diethyltoluamide), and simple aromatic compounds (SACs; phenylacetic acid, benzyl benzoate, and benzyl alcohol). Among the spore-forming species with high nematicidal activity, *Cytobacillus firmus* synthesizes all five classes of nematicidal metabolites, with a ratio over 30% of PDs, BSMHCs, and SACs. Moreover, *B. wiedmannii* ZZQ-15, *B. mycoides* ZZQ-1576, and *B. thuringiensis* ZZQ-1522, ZZQ-1524, ZZQ-1551, ZZQ-1552, and ZZQ-1553 synthesize novel cyclopeptides with potential nematicidal activity. This study provides a useful method to identify nematicidal metabolites of bacteria.

## 1. Introduction

Plant-parasitic nematodes [1] and plant pathogenic bacteria [2] and fungi [3] damage crops very heavily around the world. Synthetic chemical compounds are a common strategy for controlling plant parasitic nematodes. However, widespread use of these man-made chemical nematicides is very harmful to the environment and human health, reducing their use significantly for controlling nematodes in agriculture and forestry [4]. Thus, environmentally friendly alternative strategies are demanded. Biological agents include live organisms, and their metabolic products are effective solution to control plant-parasitic nematodes. Nematicidal secondary metabolites produced by fungi and bacteria consist of alkaloids, peptide compounds, terpenoids, macrolide compounds, oxygen heterocyclic and benzo compounds, quinones, aliphatic compounds, simple aromatic compounds, and sterols [5]. However, less excavation of nematicidal metabolites from bacteria across families at a large scale have been carried out.

In the past decades, research on the control of nematodes has increasingly focused on spore-forming Bacilli, primarily because of their diverse insecticidal and/or nematicidal factors, including secondary metabolites such as thuringiensin and trans-aconitic acid from *Bacillus thuringiensis* [6],[7]. Moreover, secondary metabolites with nematicidal activity from *Bacillus* or associated with *Bacillus* pathogenesis against nematodes have been researched [8]–[10]. Our previous work proved that spore-forming Bacilli is an excellent reservoir of nematicidal bacterial resources [11] and a good biomaterial for studying population evolution between bacteria and hosts or target pathogens, such as nematodes [12]. However, some nematicidal spore-forming strains of our team, such as *Lysinibacillus sphaericus* G25-33, G25-34 and G25-62 have few nematicidal factors at the genome level [11], and the supernatant of some strains such as *Fictibacillus phosphorivorans* G25-29 and *F. arsenicus* G25-54 have nematicidal activity [13],[14], implying that secondary metabolites may contribute to their nematicidal capability.

To analyze the diversity and abundance of nematicidal metabolites in spore-forming strains across families, we decided to use metabolomics combing with search on public metabolite database. Bacterial metabolomics studies based on MS greatly improve analytical performance of metabolite identification of bacteria and biochemical details that underlie the effect of enteric community of bacteria on the host in a post-genomic era [15],[16]. Comparison of identified secondary metabolites with some metabolite databases, such as the Kyoto Encyclopedia of Genes and Genomes (KEGG) compound database (http://www.kegg.jp/kegg/compound/), can confirm specifically bioactive secondary metabolites, including nematicidal secondary metabolites.

In this study, we identified that spore-forming strains produce nine nematicidal secondary metabolites, which are combined into five classes, and high nematicidal spore-forming species produce more nematicidal metabolites. *C. firmus* as one high nematicidal spore-forming species kills nematodes with more PDs, BSMHCs and SACs. Additionally, spore-forming strains produce cyclic dipeptides, which are confirmed with nematicidal activity [17]. The results in this study demonstrated that metabolomics can be used to identify potential metabolites of bacteria across families with special activities, and the diversity and abundance of metabolites are closely related to nematicidal activity of spore-forming strains.

## 2. Results

### 2.1. Spore-forming Bacilli produce nine nematicidal secondary metabolites

In our previous research, some spore-forming strains killed nematodes with high nematicidal activity but harbored few nematicidal factors. We speculated that secondary metabolites may contribute to their nematicidal capabilities. To mine the nematicidal secondary metabolites of these strains, we detected their secreted metabolome with MALDI-TOF/TOF. The results showed that 77 spore-forming strains produced a total of 662 secondary metabolites, including 447 non-peptide compounds and 215 peptide compounds, which are composed of 170 tripeptides and 45 dipeptides (Table S1). By comparing this with the compound database of KEGG, we found that 201 of the 447 non-peptide compounds have different bioactivities, including nine nematicidal secondary metabolites, selamectin, prometon, phenylacetic acid, diethyltoluamide, diethylcarbamazine, crotamiton, benzyl benzoate, benzyl alcohol, and amodiaquine (Table S1).

**Figure 1.**
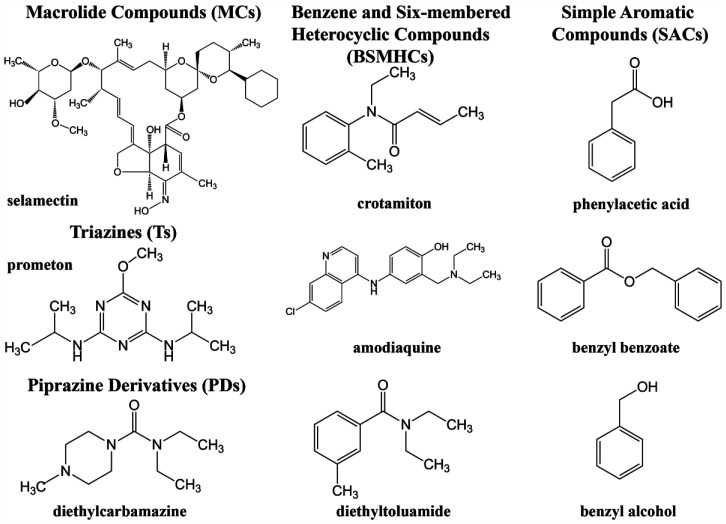
The chemical structures of nine nematicidal compounds produced by spore-forming bacteria. The nine nematicidal compounds are spread over five classes, including MCs, Ts, PDs, and bBSMHCs and SACs.

Nine nematicidal secondary metabolites can be classified into five classes based on their structures, including MCs, PDs, Ts, BSMHCs, and SACs (Figure 1). In the first class, selamectin, as an avermectin derivative, is a classical nematicide and acaricide in dogs and cats. Selamectin can activate chloride channel gates by glutamic acid during muscle synapse in parasitic nematodes and mites, leading the entry of chloride into nerve cells through this channel, causing neuromuscular paralyzation and destruction of muscle contractions, and killing them. In the second class, diethylcarbamazine, which is synthetical and kills filarials in humans, dogs, and cats, specifically. Diethylcarbamazine, an inhibitor of arachidonic acid metabolism, can make microfilaria more sensitive to innate immunity of the host. However, it is unable to kill microfilaria completely. Moreover, prometon of the third class, diethyltoluamide, crotamiton, and amodiaquine of the fourth class, and benzyl alcohol, phenylacetic acid, and benzyl benzoate of the fifth class have antiparasitic activity, including nematicidal or acaricidal activities.

**Figure 2.**
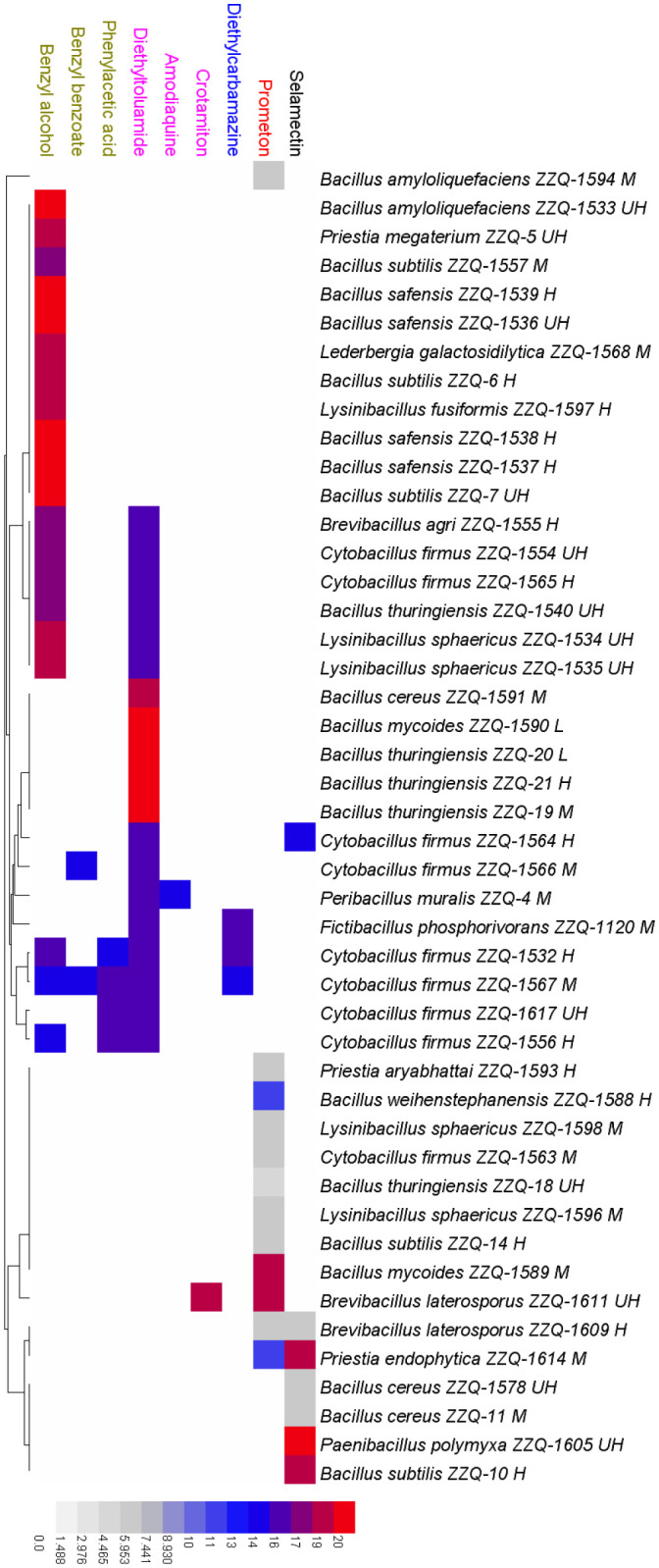
Distribution of nine nematicidal compounds across spore-forming bacteria. UH, H, M, and L after strains mean that these strains have ultra-high (mortality > 60%), high (40% < mortality ≤ 60%), medium (20% < mortality ≤ 40%) and low (0 < mortality ≤ 20%) nematicidal capability, respectively.

Nematicidal compounds show different concentrations in spore-forming strains. Among the above five class of nematicidal compounds, the concentrations of all PDs, BSMHCs, and SACs are higher than 15 (Log FC (abs) ([strain] Vs [control]), which refers to the concentration of different compounds with abs standing for absorbance), but MCs and Ts cover a large span in concentration (Figure 2). Benzyl alcohol of SACs and diethyltoluamide of BSMHCs are the two most popular nematicidal compounds produced by 20 and 19 of the 46 spore-forming strains, respectively (Figure 2 and Table S1).

### 2.2. High nematicidal spore-forming species produce more nematicidal compounds

**Figure 3.**
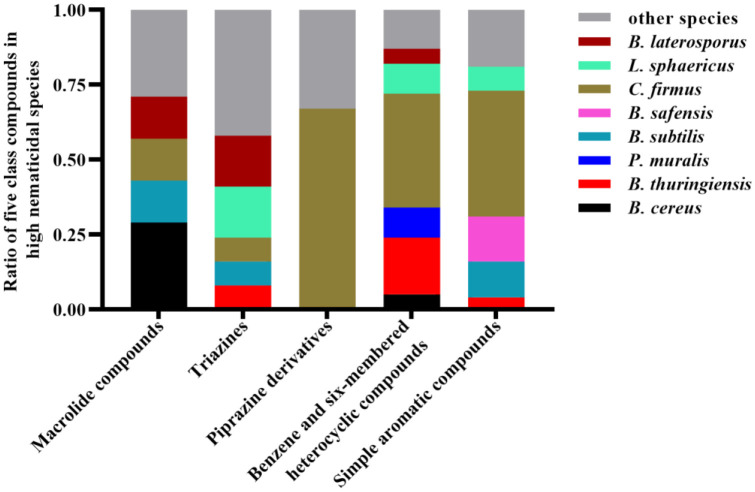
Content of five classes of nematicidal compounds in spore-forming species with high nematode-killing capability.

In our previous research, we found nine species, including *B. thuringiensis*, *B. cereus*, *B. subtilis*, *Peribacillus muralis*, *Cytobacillus firmus*, *B. toyonensis*, *L. sphaericus*, *Brevibacillus laterosporus*, and *B. brevis*, that are highly nematicidal [11]. The metabolome showed that 46 of the 77 spore-forming strains produced at least one of the above nine nematicidal secondary metabolites (Table S1). More than half of the nematicidal compounds in each class was distributed in the metabolome of high nematicidal species except *B. brevis* (Figure 3). Among the 46 strains, *L. sphaericus* ZZQ-1534 and ZZQ-1535 produced two nematicidal secondary metabolites, giving a reasonable explanation why *L. sphaericus* with highly nematicidal activity had few nematicidal factors identified at the genome level. These findings demonstrated that high nematicidal spore-forming species can produce more toxic compounds to kill nematodes.

### 2.3. *C. firmus* tend to kill nematodes with BSMHCs and SACs

Among the eight high nematicidal species in this study, *C. firmus* was the only species that could produce all five classes of nematicidal compounds (Figure 3). In these compounds, BSMHCs and SACs had a higher frequency of occurrence in *C. firmus* than other compounds (Table S1). Moreover, phenylacetic acid and benzyl benzoate of SACs exist only in metabolites of *C. firmus* (Table S1).

### 2.4. Spore-forming strains produced diverse potential nematicidal cyclic dipeptides

**Figure 4.**
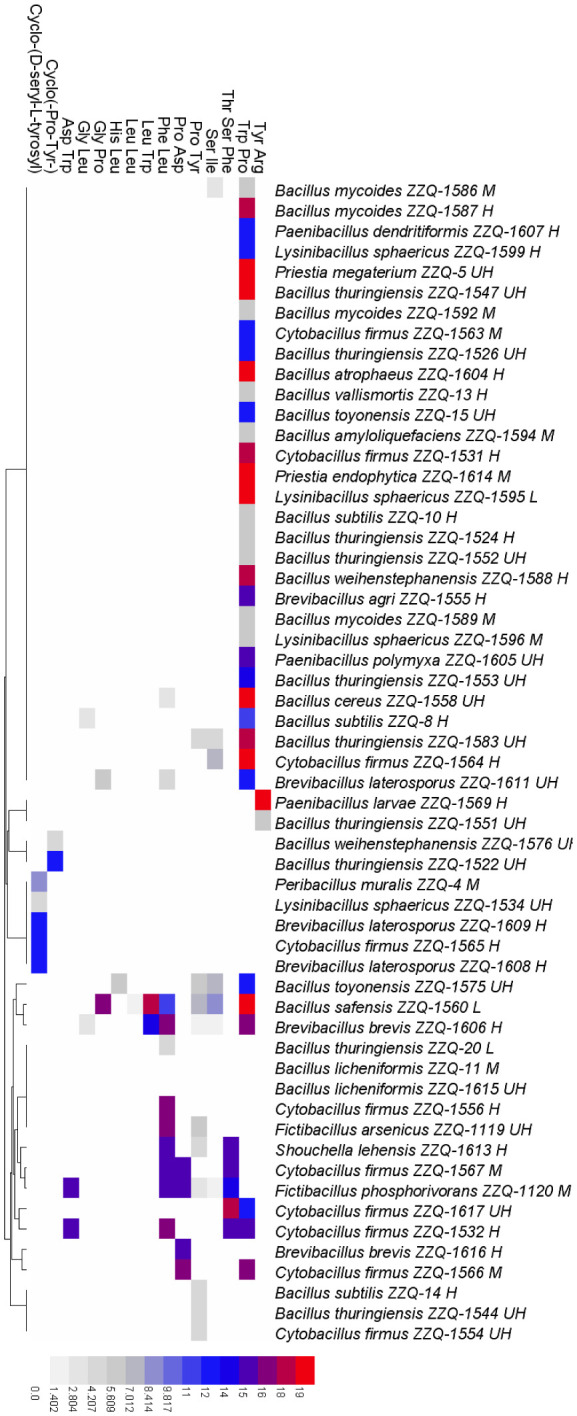
Distribution of dipeptide and tripeptide across spore-forming bacteria. Strains followed by UH, H, M, and L meant that these strains had ultra-high (mortality > 60%), high (40% < mortality ≤ 60%), medium (20% < mortality ≤ 40%), and low (0 < mortality ≤ 20%) nematicidal capability, respectively.

In addition to the non-peptide compounds, one third of the produced secondary metabolites were peptide compounds, including 47 dipeptides and 174 tripeptides (Figure 4 and Table S1). In the identified dipeptide compounds pools, there were two cyclic dipeptides (Figure 4).

Cyclic dipeptides are the simplest diketopiperazines (DKPs), which are natural six-member ring compounds usually condensed by two L-amino acids. The two cyclic dipeptides are cyclo (L-prolyl-L-tyrsyl) and cyclo (D-seryl-L-tyrosyl), both of which exist in strains of the *B. cereus* group. For example, cyclo (L-prolyl-L-tyrsyl) exists in *B. wiedmannii* ZZQ-15 and *B. mycoides* ZZQ-1576, and cyclo (D-seryl-L-tyrosyl) exists in *B. thuringiensis* ZZQ-1522, ZZQ-1524, ZZQ-1551, ZZQ-1552, and ZZQ-1553 (Figure 4). Moreover, we detected linear dipeptides of cyclo (L-leucyl-L-phenylalanyl) and cyclo (L-leucyl-L-leucyl), indicating that both patterns may exist in the secondary metabolites of spore-forming strains (Table S1). Additionally, one cyclic dipeptide cyclo (L-isoleucyl-L-proline) from *Pseudomonas putida* showed nematicidal activity [17], implying that the cyclic dipeptides may contribute to nematicidal activity of spore-forming strains because of their same diketopiperazine six-member rings.

## 3. Discussion

### 3.1. Nematicidal spore-forming strains may harbor putative gene clusters for synthesizing secondary metabolites

To understand the biosynthesis of the nine nematicidal compounds, we sequenced the genomes of 46 spore-forming bacteria and identified gene clusters with antiSMASH (http://antismash.secondarymetabolites.org/). The results showed that 46 spore-forming strains harbor 27 types of non-ribosomal peptide synthetase (NRPS) clusters, 11 polyketide synthetase (PKS) clusters, and 53 hybrid polyketide synthetase-non-ribosomal peptide synthetase (PKS/NRPS) clusters. The 91 types of gene clusters had different distributions among the genomes of 46 spore-forming strains, and there were 0–2 copies per genome for one gene cluster (Table S2). However, functions of synthetase genes in the clusters need to be confirmed, and the corresponding non-ribosomal peptides, polyketides, and hybrid polyketide synthetase-non-ribosomal peptide also need to be identified further.

### 3.2. The wide array of nematicidal metabolites detected in *C. firmus* likely explains its high nematicidal activity

*C. firmus*, as a very successful bionematicida, such as BioNem-WP developed by Agro-Green Company of Israel, effectively controls several Plant-parasitic nematodes (PPNs), such as the root-knot nematode *Meloidogyne* spp [18],[19], *Heterodera glycines* [20], *Rotylenchulus reniformis* [21], and *Belonolaimus longicaudatus* [22]. However, its nematicidal factors and mechanisms are unclear except one serine protease Sep1 of *C. firmus* DS-1 [23]. The nematicidal compounds may be important nematicidal factors of *C. firmus*, whose mechanisms need to be studied further.

### 3.3. Cyclic dipeptides may be a class of novel nematicidal metabolites from bacteria

Cyclodipeptides and their derivatives, the DKPs, constitute a large class of secondary metabolites synthesized predominantly by microorganisms. There has been considerable interest in natural DKPs in recent years because these molecules have been shown to have important and diverse biological activities, such as antibacterial [24], antifungal [25], and antitumor [26] activities. However, the biological role of DKPs in the organisms remains poorly documented.

After the formation of cyclodipeptides constituting the DKP scaffolds by an NRPS-independent pathway was discovered in *Streptomyces noursei* in 2002, 51 cyclodipeptide synthase (CDPS) have been found in different bacteria, which can produce 56 kinds of cyclodipeptides with 17 of the 20 proteinogenic amino acids [27]. Based on the sequences of known CDPS, 17 CDPS candidates have been found in 16 nematicidal spore-forming strains (data not shown). The similar DKP scaffolds of cyclodipeptides and identification of nematicidal cyclo (L-isoleucyl-L-proline) of *P. putida* [17] provoke us to identify and confirm the nematicidal activity of cyclodipeptides of these strains. With this consideration, we have cloned one CDPS from *B. paralicheniformis* ZZQ-12 and found that it can produce different cyclodipeptides with nematicidal activity (patent strain). Thus, it suggests that cyclodipeptides may be a new class of nematicidal secondary metabolites of bacteria, such as spore-forming species in this study.

## 4. Conclusions

Metabolomics studies have shown that spore-forming strains can produce five classes of nematicidal metabolites, including MCs, Ts, PDs, BSMHCs, and SACs. Among high nematicidal spore-forming species, *C. firmus* synthesizes all five classes of nematicidal metabolites, with a ratio over 30% of PDs, BSMHCs, and SACs. Phenylacetic acid and Benzyl benzoate of SACs exist only in *C. firmus*. Moreover, *B. wiedmannii* ZZQ-15, *B. mycoides* ZZQ-1576, and *B. thuringiensis* ZZQ-1522, ZZQ-1524, ZZQ-1551, ZZQ-1552, and ZZQ-1553 synthesize novel cyclopeptides with potential nematicidal activity.

## 5. Materials and methods

### 5.1. Bacterial strains and secreted metabolome preparation

Methanol and acetonitrile are both effective and commonly used organic solvents for extracting bioactive secondary metabolites from microorganisms. To prepare secreted metabolome, single colonies of all the 77 spore-forming strains were inoculated from the culture plate into 5 mL of Luria–Bertani (LB) medium with agitation. Then the overnight cultures were transferred into 250-mL ﬂasks containing 30 mL of LB medium at 1/100 (vol/vol). The ﬂasks were incubated at 30 °C for 48 h in a rotary shaker with agitation at 200 rpm to a final OD_600_. After centrifugation at 10,000 × g for 10 min at 4 °C, analytically pure absolute methanol with two times the volume of the cell-free supernatants was added. The whole mixed liquid was then placed at -20 °C for 12 h. After centrifugation at 10,000 × g for 10 min at 4 °C, sediment-free supernatants were evaporated at 37 °C until there was about 1 mL liquid left. To obtain a sufficient number of secondary metabolites covering a wide polarity range, the centrifugated supernatants were dissolved with chromatography-grade acetonitrile 10 times containing 0.1% formic acid, and then ﬁltered through a 0.22 µm membrane ﬁlter. The filtered metabolites of these strains were adjusted into OD_600_, and were deemed secreted metabolome. LB medium was the control with the same treatment, and every spore-forming strain as a condition in the following step had three replicates for three comparisons against the control.

### 5.2. Detection of the secreted metabolome by liquid chromatography quadrupole-time of ﬂight (LC-Q-TOF) mass spectrum (MS), and their analysis by the Agilent MassHunter Mass Profiler Professional (MPP) software

LC-Q-TOF MS were performed by an Agilent 1260 LC device equipped with a C_18_ reverse-phase column (100 by 1.8 mm; particle size of 3.5 µm) under chromatography detection conditions, as the ﬂow rate was decreased by 0.3 ml/min accompanied with the injection volume of 1 µL and the diode array detection at 254 nm. Q-TOF MS was carried out using a G6540A system (Agilent) equipped with a dual-source electrospray ionization (ESI) ion source in positive-ion mode. Calibration was carried out using standard references of mass 121.0509 and 922.0098. The source parameters were set as follows: gas temperature at 350 °C, gas ﬂow rate at 9 liters/min, and nebulizer stress of 40 psig. The capillary, fragmenter, skimmer, and octopole radio frequency (RF) peak voltages were set at 4,000 V, 150 V, 65 V, and 750 V, respectively, as scan source parameters.

The ions of metabolites were determined by MS with scan rates of 2.0 spectra/s. The MS scan ranges were set from m/z 50 to 2,500. Data were analyzed using Agilent MassHunter Mass Profiler Professional software. The fold change (FC) analysis of metabolites of 77 nematicidal spore-forming strains against control was performed with fold-change cut-off 2.0. The value of log2 ([sample] vs [control]) meant production of every metabolite and if they were detected; a value below zero meant the spore-forming strains did not produce the metabolite or used this metabolite to synthesize other products, and the larger values above zero indicated that the higher metabolites were produced. For the quantiﬁcation of metabolites, signiﬁcant differences between samples were evaluated using one-way analysis of variance (ANOVA) at an error probability of p < 0.05.

MPP is a chemometrics platform designed to mine the complex information content of mass-spectrometric data. Primarily used in MS-based differential analysis, it uncovers relationships among two or more sample groups and their variables. MPP offers advanced statistical analysis and visualization tools for GC/MS, LC/MS, CE/MS, and ICP-MS data, and is the only platform that integrates compound identification/annotation with integrated pathway analysis for metabolomics and proteomics research. MPP does not deliver any “official certification” for Level 1 (confirmed identification) or Level 2 (putative identification), yet it fully supports every analytical step required to achieve Level 1 and Level 2 assignments from LC-Q-TOF data with the integrated database(s), reference standards, and quality criteria. Accordingly, all compounds reported herein are considered confidently identified.

### 5.3. Testing of nematicidal activity

Nematicidal activity of selected spore-forming strains against *Caenorhabditis elegans* in this study were determined according to another [11].

## Use of AI tools declaration

The authors declare they have not used Artificial Intelligence (AI) tools in the creation of this article.




